# Correction: Ke et al. A Comprehensive Investigation into the Crystallology, Molecule, and Quantum Chemistry Properties of Two New Hydrous Long-Chain Dibasic Ammonium Salts C_n_H_2n+8_N_2_O_6_ (n = 35 and 37). *Int. J. Mol. Sci.* 2023, *24*, 5467

**DOI:** 10.3390/ijms26010091

**Published:** 2024-12-26

**Authors:** Zengbo Ke, Xinhui Fan, Youying Di, Fengying Chen, Xi Han, Ke Yang, Bing Li

**Affiliations:** 1 School of Materials Science and Chemical Engineering, Xi’an Technological University, Xi’an 710021, China; 2 College of Chemical Engineering and Modern Materials, Shaanxi Key Laboratory of Comprehensive Utilization of Tailings Resources, Shangluo University, Shangluo 726000, China

The journal’s Editorial Office and Editorial Board are jointly issuing a resolution and removal of the *Journal Notice* linked to this article [1], as well as an update to the original publication. Following concerns raised about the integrity of one of the peer-reviews, the Editorial Office has conducted a post-publication peer-review of this article. This process included the recruitment of a new independent reviewer and was supervised by the original Academic Editor to ensure full compliance with MDPI’s Editorial Process (https://www.mdpi.com/editorial_process).

As a result of this process, the Academic Editor and the authors have agreed to update the following aspect of this publication:

- Reviewer report 1 was removed from the peer review record and the new report was uploaded instead (https://www.mdpi.com/1422-0067/24/6/5467/review_report).

Based on the new review report, the following corrections have been made to the original publication:

Introduction, 2.3, 2.4.1–3 in the Results and Discussions sections, 3.1 in the Materials Methods section and References 46 and 47 were corrected.

With this update, the Academic Editor is satisfied that the Editorial Process relating to this article has been completed as per MDPI’s Editorial Process policy. The Editorial Office would like to thank the authors for their collaboration during this process. 
