# Supplementary material for: A Comprehensive Investigation into the Crystallology, Molecule, and Quantum Chemistry Properties of Two New Hydrous Long-Chain Dibasic Ammonium Salts CnH2n+8N2O6 (n = 35 and 37)
Source: Int J Mol Sci. 2023 Mar 13;24(6):5467. doi: 10.3390/ijms24065467 (PMC10052139; doi:10.3390/ijms24065467)
Supplement: Supplementary file 1 [file ijms-24-05467-s001.zip › ijms-2252641-supplementary.pdf]

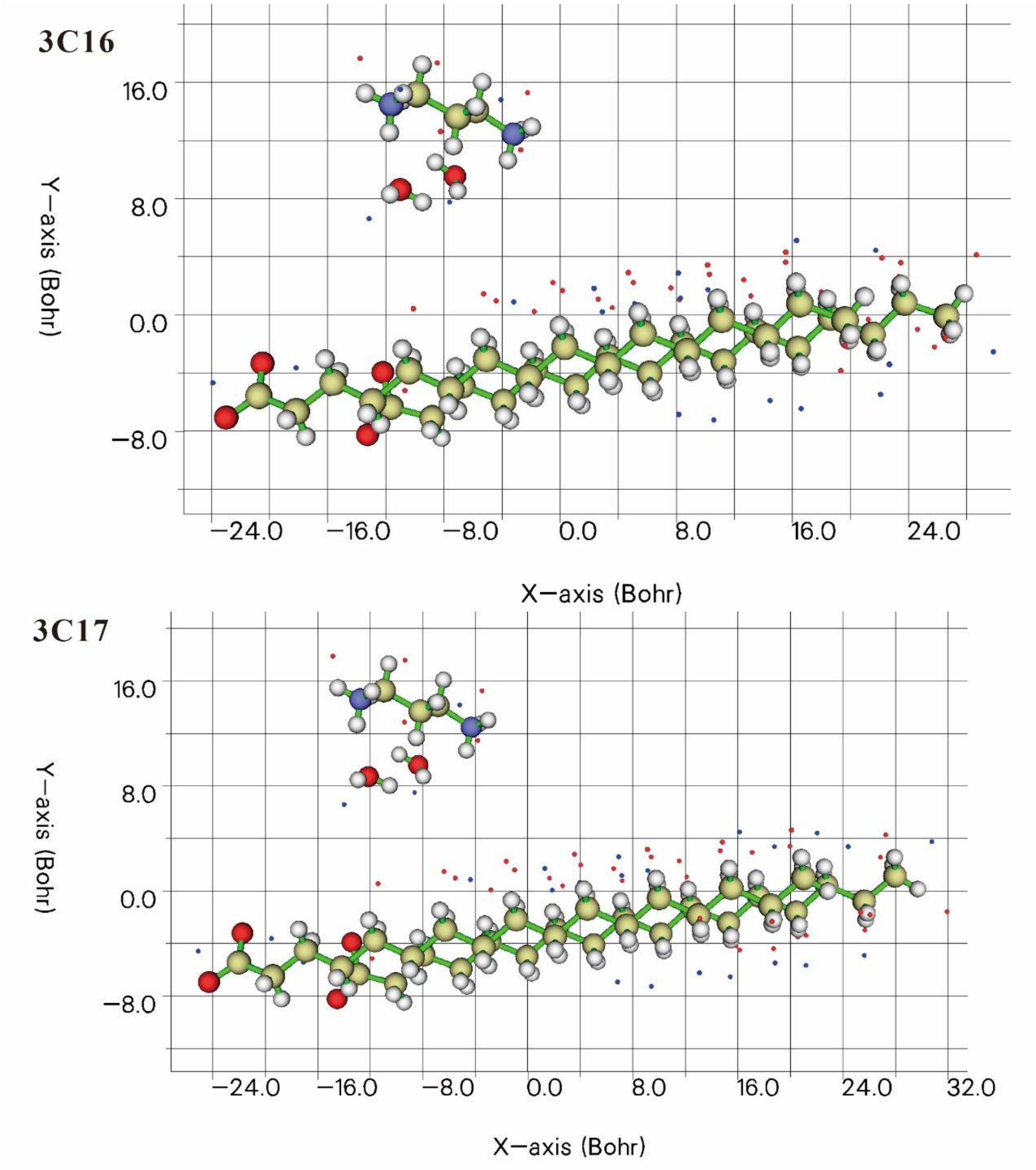

Figure S1. Molecular surface electrostatic potential distribution of 3C16 and 3C17.

Table S1. Void volumes, surface areas and void volumes as a percentage of total unit cell volume for different choices of isovalue for 3C16 and 3C17.

Molecule	Isovalue/au	Volume/Å^3	Surface area/Å^2	% of cell volume
3C16	0.0003	0.50	5.81	0.03
	0.0005	10.44	64.82	0.53
	0.0008	37.65	167.71	1.92
	0.001	57.51	228.3	2.93
	0.002	214.46	785.67	10.94
3C17	0.0003	0.17	2.09	0.01
	0.0005	5.07	38.55	0.24
	0.0008	30.3	157.41	1.45
	0.001	52.49	250.69	2.52
	0.002	248.87	912.08	11.93

Table S2. Hirshfeld contact surfaces and derived ‘random contact’ and ‘enrichment ratios’ for the 3C16 crystal.

Atoms	H	O	C	N
H	79.4	8.8	0.4	Contacts
O	10.6	0.2	_	(%)
C	0.5	_	_	_
N	0	_	_	_
Surface(%)	90.5	9.0	0.4	_
H	81.9	7.4	0.2	Contacts
O	8.9	0.8	_	Random
C	0.6	_	_	(%)
N	0	_	_	_
H	0.97	1.19	2.0	Enrichment
O	1.19	0.25	_	_
C	0.83	_	_	_
N	0	_	_	_

Table S3. Hirshfeld contact surfaces and derived 'random contact' and 'enrichment ratios' for the 3C17 crystal.

Atoms	H	O	C	N
H	80.0	8.5	0.4	Contacts
O	10.3	0.2	_	(%)
C	0.4	_	_	_
N	0	_	_	_
Surface(%)	90.7	8.8	0.5	_
H	82.3	7.2	0.45	Contacts
O	8.7	0.8	_	Random
C	0.45	_	_	(%)
N	0	_	_	_
H	0.97	1.18	0.89	Enrichment
O	1.18	0.25	_	_
C	0.89	_	_	_
N	_	_	_	_

Table S4. The electrostatic potential distribution on the molecular surface of 3C16 (the data with * indicates the extreme point).

Number of surface minima	a.u.	eV	kcal/mol	X/Y/Z coordinate(Angstrom)		
1	-0.16453	-4.47702	-103.243	-11.2868	-2.02493	-0.23548
2	-0.17027	-4.63317	-106.844	-8.72145	-1.60206	-1.2858
***3**	**-0.18087**	**-4.92175**	**-113.498**	**-7.75474**	**-2.55052**	**-2.37115**
4	-0.02438	-0.66343	-15.299	-6.35988	3.411512	-1.94315
5	0.154834	4.213252	97.15999	-4.65438	7.243617	4.013048
6	-0.02839	-0.77248	-17.8137	-3.8894	4.060429	-5.12827
7	0.131806	3.586613	82.70933	-1.67138	7.080439	2.231581
8	0.008044	0.218889	5.047709	-1.29557	0.681204	1.492379
9	0.005605	0.152513	3.517026	0.000434	0.001186	-0.60823
10	0.013988	0.380633	8.777612	1.270841	1.117332	1.878849
11	0.013392	0.364421	8.403752	1.533637	0.31979	0.159183
12	0.005996	0.163173	3.762859	2.637313	0.520624	-2.45775
13	-0.0015	-0.04087	-0.9424	3.929375	-2.81683	2.71099
14	0.013188	0.358868	8.275706	3.939541	1.602784	2.050828
15	0.015495	0.421627	9.722963	4.078786	0.770223	0.428281
16	0.007731	0.210363	4.851096	5.118993	1.002794	-2.08478
17	-0.00279	-0.07603	-1.75324	5.270422	-3.3381	-1.28522
18	0.001694	0.046096	1.062991	6.761744	-2.35995	2.986289
19	0.010461	0.284664	6.564516	7.662142	2.63332	2.302381
20	-0.00043	-0.01171	-0.27009	8.177383	-2.94931	-1.1422
21	0.00272	0.07402	1.706946	10.46661	-1.22758	3.099985
22	0.000575	0.015647	0.360817	10.79381	-2.45708	-0.8996
23	0.004376	0.119074	2.745905	10.71045	2.33813	-1.40719
24	-0.00049	-0.01324	-0.30526	14.46594	-1.02014	-0.51419
**Number of surface maxima**	**a.u.**	**eV**	**kcal/mol**	**X/Y/Zcoordinate(Angstrom)**		
1	0.174084	4.737079	109.2397	-6.24712	8.491867	1.227791
2	-0.0646	-1.75798	-40.54	-4.96487	-2.28594	-0.26814
3	-0.00379	-0.10307	-2.37695	-4.37301	0.499905	2.836129
4	0.163501	4.449091	102.5986	-3.95133	8.562197	-0.84213
5	0.168485	4.584708	105.726	-3.54038	6.048605	2.611593
6	0.014768	0.401862	9.267171	-2.17544	0.961225	2.88742
7	0.009981	0.271603	6.263316	-1.90906	0.695334	0.769408
8	0.171845	4.676127	107.8341	-1.11462	5.5864	0.633291
***9**	**0.176002**	**4.789245**	**110.4427**	**-1.01468**	**7.709102**	**-2.40349**
10	0.007469	0.20325	4.68706	-0.7266	0.301428	-0.90077
11	0.02187	0.59511	13.72357	-0.00455	1.318212	3.097962
12	0.020062	0.545926	12.58937	0.249353	1.020814	0.699309

13	0.016749	0.455752	10.5099	1.397522	0.714272	-0.63209
14	0.008902	0.242232	5.586006	1.878454	0.35995	-3.47918
15	0.022175	0.603413	13.91506	2.339536	1.63042	3.454155
16	0.02077	0.565185	13.0335	2.544838	1.286011	0.626853
17	0.017867	0.486175	11.21146	3.79263	1.090075	-0.38668
18	0.013111	0.356769	8.227294	4.20413	0.624539	-3.5348
19	0.020176	0.54902	12.66071	4.763124	1.869393	3.927882
20	0.018522	0.504011	11.62277	5.015043	1.548659	0.572946
21	0.016798	0.457102	10.54103	6.177074	1.367294	0.016231
22	0.014252	0.387822	8.9434	6.696013	0.756833	-3.57825
23	0.018569	0.505275	11.65193	7.137496	2.261866	4.170209
24	0.016862	0.458836	10.58101	7.487066	1.947482	0.644759
25	0.010723	0.291781	6.728637	8.569841	-1.60058	-3.35366
26	0.015556	0.4233	9.761539	8.838187	-0.55271	4.94698
27	0.013597	0.369987	8.532115	9.128449	0.916611	-3.49573
28	0.013782	0.37502	8.64817	9.206488	-1.54296	1.176996
29	0.01823	0.496064	11.43952	10.31732	2.08734	2.568623
30	0.011121	0.302609	6.978333	10.68749	-0.03747	-3.00227
31	0.01146	0.311839	7.191191	11.21573	-1.20705	-3.09451
32	0.014313	0.389483	8.98171	11.19676	1.932719	0.807084
33	0.013069	0.355635	8.201142	11.76762	1.432635	-3.22327
34	0.012543	0.341305	7.870698	12.33976	-0.36814	-2.74104
35	0.013009	0.35398	8.162988	12.53867	-0.46368	1.305257
36	0.012624	0.343527	7.921918	12.91783	-0.97854	-2.75194
37	0.015191	0.413358	9.532272	13.98223	2.190865	-0.89459

Table S5. The electrostatic potential distribution on the molecular surface of 3C17 (the data with * indicates the extreme point).

Number of surface minima	a.u.	eV	kcal/mol	X/Y/Z coordinate(Angstrom)		
1	-0.1636	-4.45181	-102.661	-11.9085	-1.99734	-0.24348
2	-0.1685	-4.58513	-105.736	-9.40271	-1.59916	-1.29443
***3**	**-0.17935**	**-4.88029**	**-112.542**	**-8.38295**	**-2.55955**	**-2.39748**
4	-0.02899	-0.78883	-18.1908	-6.79626	3.392991	-2.03949
5	-0.03213	-0.87436	-20.1633	-4.41309	3.92474	-5.06832
6	0.132711	3.611239	83.27722	-2.19178	6.814799	2.19633
7	0.007183	0.195463	4.507477	-1.85527	0.651875	1.34083
8	0.013252	0.360613	8.315941	0.79948	1.058245	1.744755
9	0.012426	0.338122	7.797281	1.04878	0.244777	0.143264
10	0.005214	0.141886	3.271971	2.075822	0.460598	-2.52293
11	-0.00153	-0.04151	-0.95732	3.349939	-2.88238	2.710862
12	0.012335	0.335653	7.740345	3.41711	1.473553	1.984267
13	0.014612	0.397623	9.169403	3.567028	0.785782	0.521668
14	0.007135	0.194164	4.477527	4.62557	0.913192	-2.23322
15	-0.00323	-0.08788	-2.02662	4.720756	-3.37861	-1.33302
16	0.001021	0.027785	0.640734	6.195344	-2.54961	2.910019
17	-0.0003	-0.00818	-0.18872	7.630265	-3.00132	-1.07379
18	0.008825	0.240127	5.537476	7.654374	2.347407	2.249425
19	0.002344	0.063774	1.470674	8.812201	-2.19961	3.029691
20	0.005134	0.13971	3.221789	9.359405	1.814437	-1.83944
21	0.000274	0.007457	0.171956	10.41686	-2.5685	-0.93143
22	0.003978	0.108234	2.495947	10.94275	2.321264	-1.72689
23	0.004883	0.132862	3.063862	11.38995	1.837437	2.798236
24	0.00035	0.009532	0.219819	12.52812	-2.18248	-0.67011
25	0.001407	0.038292	0.88303	15.11443	2.002805	-1.16266
**Number of surface maxima**	**a.u.**	**eV**	**kcal/mol**	**X/Y/Z coordinate(Angstrom)**		
***1**	**0.176608**	**4.805753**	**110.8234**	**-6.76955**	**8.628194**	**1.322795**
2	-0.06353	-1.72885	-39.8682	-5.55241	-2.26965	-0.25296
3	-0.00364	-0.09907	-2.28449	-4.99689	0.544983	2.830868
4	0.163558	4.450638	102.6342	-4.38887	8.69457	-0.79437
5	0.170789	4.647402	107.1717	-4.08265	6.180803	2.686758
6	0.014863	0.40445	9.326856	-2.69532	0.977395	2.901049
7	0.008834	0.240387	5.543464	-2.43156	0.69611	0.793618
8	0.170751	4.646375	107.148	-1.63276	5.651626	0.61141
9	0.174342	4.744086	109.4013	-1.63689	7.709999	-2.51374
10	0.006046	0.164511	3.793715	-1.22933	0.221964	-0.80932
11	0.021766	0.592286	13.65846	-0.51993	1.327431	3.065997

12	0.019051	0.518403	11.95468	-0.30384	0.989092	0.661324
13	0.015987	0.435018	10.03176	0.935921	0.66741	-0.63873
14	0.007909	0.215207	4.962794	1.408729	0.297469	-3.50218
15	0.021901	0.595958	13.74313	1.851407	1.5785	3.453887
16	0.019796	0.53869	12.4225	2.083969	1.173466	0.506253
17	0.017339	0.471827	10.8806	3.295408	1.019625	-0.40342
18	0.012472	0.339367	7.826008	3.705203	0.492148	-3.65773
19	0.0198	0.538797	12.42496	4.34317	1.746123	3.899713
20	0.017859	0.48597	11.20674	4.626758	1.450578	0.520384
21	0.016343	0.444719	10.25548	5.684332	1.302464	-0.12283
22	0.013652	0.371493	8.566835	6.071878	-0.56799	4.761854
23	0.013514	0.367724	8.479921	6.196842	0.638312	-3.71757
24	0.017652	0.480348	11.07711	6.886938	2.002906	4.156496
25	0.015739	0.428274	9.87624	7.127111	1.673343	0.546919
26	0.011124	0.30271	6.980666	7.79932	-1.88915	0.829915
27	0.010453	0.284429	6.559105	8.077398	-1.99989	-3.40163
28	0.015267	0.415426	9.579951	8.311999	1.615191	0.196835
29	0.013359	0.363512	8.382801	8.484407	-0.6657	4.946163
30	0.013244	0.360375	8.310448	8.718766	0.760336	-3.68674
31	0.011355	0.30899	7.125477	9.001618	-1.83473	0.980333
32	0.017944	0.488273	11.25985	9.238059	2.406352	4.163059
33	0.016078	0.437508	10.08918	9.627346	1.840446	0.643807
34	0.011265	0.306531	7.068774	10.15856	-1.34879	1.054332
35	0.010909	0.296838	6.845255	10.41396	-1.65276	-3.23827
36	0.012608	0.343073	7.911455	11.39364	1.055675	-3.5221
37	0.015235	0.414555	9.559876	11.73779	-0.41798	3.296805
38	0.011526	0.313649	7.232919	12.41312	-0.59844	1.258983
39	0.010665	0.290214	6.6925	12.92971	-1.37426	-2.92657
40	0.014306	0.389288	8.977193	13.09965	2.255691	0.572747
41	0.013745	0.374019	8.62508	13.57756	1.403746	-3.22823
42	0.013283	0.361453	8.335319	15.52388	-0.56621	-0.42381
